# The effects of diagnosis-related groups payment on hospital healthcare in China: a systematic review

**DOI:** 10.1186/s12913-020-4957-5

**Published:** 2020-02-12

**Authors:** Kun Zou, Hong-Ying Li, Die Zhou, Zai-Jun Liao

**Affiliations:** Department of Medical Records and Statistics, Sichuan Academy of Medical Sciences & Sichuan Provincial People’s Hospital, Affiliated Hospital of University of Electronic Science and Technology of China, School of Medicine, University of Electronic Science and Technology of China, Chengdu, China

**Keywords:** Diagnosis-related groups, Hospital payment reform, Effects, Hospital healthcare, China, Systematic review

## Abstract

**Background:**

There has been a growing interest in using diagnosis-related groups (DRGs) payment to reimburse inpatient care worldwide. But its effects on healthcare and health outcomes are controversial, and the evidence from low- and middle- income countries (LMICs) is especially scarce. The objective of this study is to evaluate the effects of DRGs payment on healthcare and health outcomes in China.

**Method:**

A systematic review was conducted. We searched literature databases of PubMed, Cochrane Library, EMBASE, Web of Science, Chinese National Knowledge Infrastructure and SinoMed for empirical studies examining the effects of DRGs payment on healthcare in mainland China. We performed a narrative synthesis of outcomes regarding expenditure, efficiency, quality and equity of healthcare, and assessed the quality of evidence.

**Results:**

Twenty-three publications representing thirteen DRGs payment studies were included, including six controlled before after studies, two interrupted time series studies and five uncontrolled before-after studies. All studies compared DRGs payment to fee-for-service, with or without an overall budget, in settings of tertiary (7), secondary (7) and primary care (1). The involved participants varied from specific groups to all inpatients. DRGs payment mildly reduced the length of stay. Impairment of equity of healthcare was consistently reported, especially for patients exempted from DRGs payment, including: patient selection, cost-shifting and inferior quality of healthcare. However, findings on total expenditure, out of pocket payment (OOP) and quality of healthcare were inconsistent. The quality of the evidence was generally low or very low due to the study design and potential risk of bias of included studies.

**Conclusion:**

DRGs payment may mildly improve the efficiency but impair the equity and quality of healthcare, especially for patients exempted from this payment scheme, and may cause up-coding of medical records. However, DRGs payment may or may not contain the total expenditure or OOP, depending on the components design of the payment. Policymakers should very carefully consider each component of DRGs payment design against policy goals. Well-designed randomised trials or comparative studies are warranted to consolidate the evidence of the effects of DRGs payment on healthcare and health outcomes in LMICs to inform policymaking.

## Background

In medicine, the provider payment method is an important measure to allocate healthcare resources, to influence behaviours of health providers, and to achieve goals of the health system [1]. Originated from the United State at 1970s [2], the diagnosis-related groups (DRGs) payment method is adapted by growing number of countries and regions for inpatient care, to contain the expenditure and increase the transparency, efficiency and quality of healthcare [3–5]. Previous studies suggested that DRGs payment may mildly increase the efficiency and contain cost, with no major adverse effects on quality of healthcare under close monitoring [1, 6]. However, the evidence comes mainly from high income countries (HICs), those from low- and middle-income countries (LMICs) is urgently needed but scarce [5, 6].

In the most recent health reform, China has made great achievement toward universal health coverage (UHC) through expanding national health insurance schemes to up to 95% of the population, which reimburse about 50–70% of healthcare expenditure of the insured [7]. In the new round of health reform, the provider payment reform is one of the core measurs, in which DRGs payment is considered as an important alternative of the conventional fee-for-service (FFS) payment method and a vital component in the mixed payment system for hospitals [8]. As public hospitals in China largely rely on themselves to make revenue through providing services rather than public funds or government budgets, they may be more sensitive to financial incentives introduced by provider payment reform [9, 10]. In the last decade, several experiments of DRGs payment have been conducted in mainland China. However, the reported effects of DRGs payment on healthcare were mixed, and relevant evidence has not been systematically reviewed [11–15].

### Description of the condition

This review focused on inpatient care, which usually occurred in the setting of tertiary or secondary care. However, unlike many other countries, primary care facilities (mainly township hospitals in rural China and community health centres in urban areas) are also important providers of inpatient care in China [7], thus were also considered in this review.

### Description of the intervention

The DRGs payment system has two fundamental components. The first is the grouping logics which classify tens of thousands of inpatient services into a limited number of groups (often hundreds or around a thousand) based on the similarity of diagnoses and treatments patients received and relevant resources used. The second is the fixed price of each group defined by its average cost, which forms the base rate of reimbursement to healthcare providers for services they provide to each inpatient, with or without adjustment to regional economic status or hospital characteristics [5, 16]. DRGs payment can be applied at regional, institutional (hospital) or individual (physician) level. The latter is considered to be stronger in incentivizing behaviour changes of healthcare providers.

### How the intervention might work?

DRGs payment is a type of prospective case payment method, which is often implemented by the health insurance bodies, either public or private. Since the reimbursement price of a case (including the whole services provided to a patient) is defined previously by the average cost of the group it is classified into, mechanically it shifts the certain financial risk of healthcare from patients or insurers to health providers. Therefore, it offers health providers incentives to contain the cost of healthcare for each inpatient by reducing unnecessary services, shortening the length of stay (LOS) in hospitals and increasing the number of treated patients in a certain period, thus could increase the efficiency of healthcare and obtain more profit [17]. However, there may be some unwanted effects of the payment method. It is concerned that patients may be undertreated (denied of optimal services), discharged earlier (bloody discharge), or selected depending on the profit a hospital would make (patient selection), because hospitals are under pressure of cost containing [18]. It is also concerned that where out-of-pocket payment (OOP) from patients is needed, patients may be charged more on the OOP or the overall payment, especially when they are exempted from the DRGs payment scheme (cost-shifting). All these unwanted effects may impair the quality or the equity of healthcare [17]. Thus, it is suggested that measures to monitor, assure and improve quality and equity of healthcare are warranted in the design of DRGs payment policy [19].

### Why it is important to do this review?

There is a gap between current evidence of the effects of DRGs payment on healthcare and what is needed to inform health policy making in LMICs. As far as we know, most of the evidence regarding DRGs payment came from HICs. While it is important and valuable, it may be difficult to adapt it directly to LMICs. Comparing to HICs, LMICs often have more challenges and difficulties to implement DRGs payment. They usually have fewer resources, weaker health workforce and inadequate medical supplying [20, 21]. Additionally, applying DRGs payment often requires strong management capability of governing bodies, sophisticated and coordinated accounting and financing systems and health information systems, which are also often inadequate in many LMICs [21, 22]. Thus, evidence of DRGs payment directly comes from LMICs may be more relevant and helpful to inform health policy making in these counties [6, 19].

However, previous relevant systematic reviews (SR) either only summarised the progress of DRGs payment in LMICs but did not examine its effects on healthcare [5], or did not focus on DRGs payment specifically, thus included very limited number of relevant studies [23], or investigated the simplified case payment without core components of DRGs logics and the pricing system, thus may not be the “real” DRGs payment per se [24]. However, the emerging experiments of DRGs payment in China in recent years have offered us a good opportunity to consolidate the evidence and shed lights on the potential benefits and harms of DRGs payment, which could help policy making in health reform in China and other LMICs in similar scenario. Therefore, the objective of this study was to summarise the evidence of the effects of DRGs payment on healthcare and patient health outcomes in mainland China.

## Methods

A SR with narrative synthesis of evidence was conducted and reported according to the Preferred Reporting Items for Systematic Reviews and Meta-Analyses (PRISMA) [25].

### Inclusion criteria of studies

We included randomized controlled trials (RCTs), non-randomised clinical trials (NRCTs), controlled before-after (CBA) studies, interrupted time series studies (ITS) and uncontrolled before-after studies (BA) using the definitions of study designs of EPOC [26, 27]. The DRGs payment can applied at regional, institutional or individual level, in tertiary, secondary or primary care settings to reimburse inpatient services, with no restriction on types or version of DRGs systems, comparing to any other payment methods such as FFS, salary, global budget, per-diem payment or their combination. To be included, the study needed to report outcomes of health expenditure (e.g. health expenditure per admission (EPA), OOP per admission), efficiency (e.g. LOS), quality, equity of healthcare or patient health outcomes (as defined by the primary study); and had to have been conducted in mainland China, for healthcare systems in other territories of China were different from the mainland’s. The languages were restricted to Chinese and English, as research from China were mainly published in the two languages.

### Literature search and study selection

We searched electronic databases of PubMed, Cochrane Library, EMBASE, Web of Science, Chinese National Knowledge Infrastructure (CNKI) and SinoMed from their initiatives to May 2018. The search strategy was firstly designed in PubMed using MeSH and free-texts (appendix search strategy), and adapted for other databases. Chinese terms equivalent to English terms were used in search of Chinese literature databases. References of included studies and relevant reviews were scanned for potential eligible studies. Study selection was conducted by two reviewers (KZ, DZ or ZJL) independently according to the study inclusion and exclusion criteria, first by scanning titles and abstracts and later by reading full texts. Disagreements were resolved by discussion.

### Data extraction and quality assessment

Data extraction was performed by one reviewer (KZ) using pre-defined data extraction form and checked by an assistant (XYL) independently. Disagreements were resolved by discussion. Data extracted included first author, publication year, study location, setting, study design, study period, type of participants, type of intervention, type of control, and outcomes. Methodological quality of included studies was assessed using modified Newcastle-Ottawa Scale (NOS) [28]. The certainty of evidence of each outcome (the extent of our confidence in the estimate of effect across studies) was evaluated using the GRADE approach [29].

### Data synthesis

We aimed to conduct meta-analysis but it was not applicable due to the diversity of study design, intervention and outcomes reported across included studies. Thus, we summarised the characteristics of included studies and performed narrative synthesis of evidence regarding the effects of DRGs payment on expenditure, efficiency, quality and equality of healthcare in mainland China. When more than one publication reported the same DRGs experiment, the one with the best methodological merits and most complete outcomes was chosen for the data synthesis. Sensitivity analysis was performed by only including primary studies with high methodological quality (low risk of bias) [30].

## Results

### Study selection

**Fig. 1:**
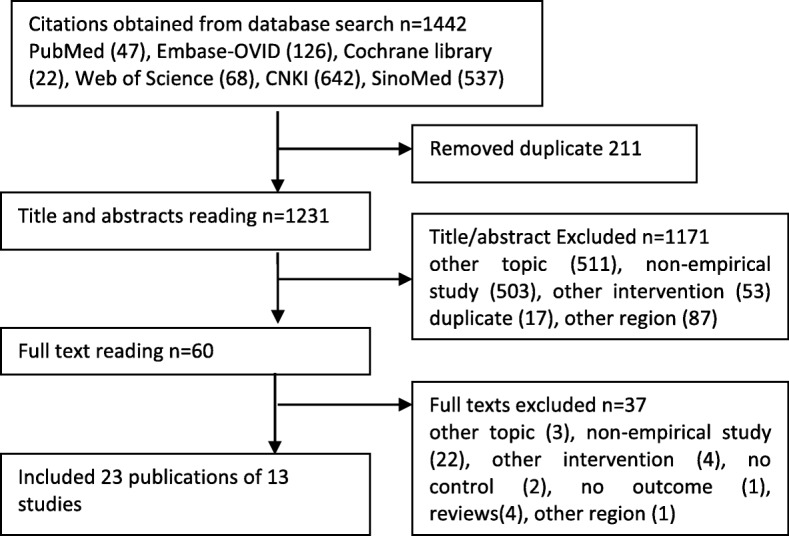
Flow chart of study selection

In total, 1442 citations were identified from the systematic literature search. After removing duplicates, the titles and abstracts of 1231 citations were scanned against inclusion criteria, among which 1171 were excluded. The full text of 60 potentially eligible citations were obtained and read and 37 citations were excluded. Finally, 23 publications representing 13 studies were included, among which 4 studies were published in English and 9 were in Chinese (Fig. 1) [11–15, 31–48].

### Characteristics of included studies

Among the thirteen studies, four were conducted in Beijing [11, 12, 43, 45], four in Yunnan province [44, 46–48], and one each in Heilongjiang Province (Harbin) [39], Guangxi province [40], Tianjin [31], Shanghai [13] and Hunan province (Changsha) [14]. Six studies were CBA [11–14, 43, 45], two were ITS [39, 41], and five were BA [31, 44, 46–48]. Among them, six studies were conducted in tertiary hospitals [11–13, 31, 43, 48], six in secondary (county) hospitals [39, 41, 44–47], while one involved primary, secondary and tertiary hospitals [14]. The participants included inpatient with urban employee medical insurance (UEMI), urban or rural resident medical insurance (URMI or RRMI), new rural cooperative medical insurance (NRCMI) or maternity insurance. All studies compared DRGs payment with FFS payment methods, with or without global budget. There were considerable variations in the components of DRGs payment policy design. All of the studies used medical records, administrative data or insurance claim data. Outcomes of EPA, OOP per admission, LOS, quality of healthcare, equity of healthcare and up-coding behaviour were reported in 13, 5, 11, 5, 4 and 2 studies, respectively (Table 1).

**Table 1:** Characteristics of included primary studies

No	Location/Study	Study design	Study period/Setting^a^	Participant/sample size, intervention vs. control	Disease categories/service specialities	Pilot	Control	Statistical test	Outcomes
1	Shanghai/Zhang 2010 [13]	CBA	2004–2005/Tertiary A hospital	Inpatient with Shanghai medical insurance/14,000 overall	15 targeted diseases, detail unreported	DRGs payment for insured patients in 1 hospital	FFS payment for uninsured patients in the same hospital	DID analysis, DDD analysis, regression analysis	1) expenditure per admission,2) length of stay, 3) equity of above indicators between insured and uninsured patients
3	Beijing/Jian 2015b (Jian 2015c) [11]	CBA	Jan 2010- Sep 2012/Tertiary A hospital	Inpatient with Beijing basic employee medical insurance/ 318,884 vs. 294,989	108 DRGs with CV < 0.85, detail unreported	DRGs payment, reimbursement ceiling, allowing for 5% annual increase in 6 hospitals	FFS payment in 8 hospitals	DID analysis, regression analysis	1) expenditure per admission, 2) out of pocket payment, 3) length of stay, 4) readmission, 5) equity, cost shifting and patient selection
4	Beijing/Zhang 2015 (Hu 2013, Hu 2014, Wu 2013, Song 2014, Jian 2015a, Tian 2015) [43]	CBA	2012–2013/Tertiary A hospital	Inpatient with urban employee medical insurance/118,091 vs. 120,427	108 DRGs with CV < 0.85, detail unreported	DRGs payment under global budget in 6 hospitals	FFS payment under global budget in 8 hospitals	Comparison of means before and after pilot, no formal statistical test	1) expenditure per admission, 2) out of pocket payment,3) length of stay, 4) 2 weeks readmission, 5) patient selection
9	Changsha, Hunan province/Zhang 2016 [14]	CBA	2013/2 primary,12 secondary and 6 tertiary hospitals	Inpatient with urban employee medical insurance/75 vs. 133	32 groups, detail unreported. Only patients with uncomplicated acute appendicitis were analysed	DRGs payment in 8 hospitals	FFS payment in 12 hospitals	Comparison of means, Student’s t-test, Pearson’s chi-square test	1) expenditure per admission, 2) length of stay,3) use of antimicrobials
5	Beijing/Poon 2017 [12]	CBA	Jan 2010 - Sep 2012/Tertiary hospital	Inpatient with basic employee medical Insurance/1374 vs. unreported	108 DRGs, detail unreported. Patients with acute myocardial infarction were analysed	DRGs payment in 6 hospitals	FFS payment in 8 hospitals	DID analysis, regression analysis	1) expenditure per admission, 2) in hospital mortality, 3) length of stay, 4) prescription of optimal AMI medications at arrival
6	Beijing/Ji 2017 (Zhang LH 2015) [45]	CBA	2011–2015/Secondary hospital	Inpatient with new rural cooperative medical insurance/unreported	All groups (from 560 to 577 at 2011 and 2014)	DRGs payment in 1 hospital	FFS payment in 10 hospitals	Comparison of means, no formal statistical test	1) expenditure per admission, 2) out of pocket payment %, 3) length of stay
7	Harbin, Heilongjiang province/Wang 2015 [39]	ITS	Aug 2010- July 2012/ Secondary A hospital	Inpatient with urban and rural resident medical insurance/213 vs. 251	36 DRG groups, detail unreported. Only patients with cholecystotomy were analysed	1) DRGs payment from Aug 2008, 2) excluding transferred patient or those under deductible or above ceiling of insurance payment in 1 hospital	FFS payment in the same hospital before the reform	t test, interrupted time series analysis	1) expenditure per admission, 2) out of pocket payment, 3) length of stay, 4) floated coding practice
8	Guangxi province/Wu 2015a (Wu 2015b) [41]	ITS	Aug 2010- July 2012/ Secondary A hospital	Inpatient with urban and rural resident medical insurance underwent herniorrhaphy/131 vs. 119	36 DRG groups including 17 diseases and 19 surgical groups, detail unclear.	DRGs payment from Aug 2011 in 1 hospital	FFS payment in the same hospital before the reform	time series analysis (ARIMA)	1) expenditure per admission, 2) out of pocket payment per capita, 3) length of stay
2	Tianjin/Li 2012 [31]	BA	2006–2012/Tertiary A hospital	Inpatient with maternity medical insurance/ 3232 vs. 5712	hospital delivery	DRGs payment from July 2009 in 1 hospital	FFS payment before July 2009 in the same hospital	Comparison of means (Mann-Whitney U test), and rate (chi square test)	1) expenditure per admission,2) length of stay, 3) caesarean rate
10	Lufeng, Yunnan province/Peng 2016 (Li 2013) [44]	BA	2012–2013/County (secondary) hospital	Inpatient with new rural cooperative medical insurance/35272 vs.32369	All patients (432 groups)	DRGs payment from Oct 2012, top 3% patients with highest expense paid by FFS payment, and penalty for readmission within 7 days in 3 hospitals	FFS payment in the same hospitals before the reform	Comparison of means before and after the pilot, no formal test	1) expenditure per admission, 2) length of stay
11	Xiangyun, Yunnan province/Peng 2017 [46]	BA	2014–2015/County (secondary) hospital	Inpatient with new rural cooperative medical insurance/19,479 vs. unreported	All patients (434 groups at 2014, 304 groups at 2015)	DRGs payment in 1 hospital from August, 2014	FFS payment in the same hospital before the reform	Comparison of means, before (2014) and after the pilot (2015), no formal test	1) expenditure per admission, 2) length of stay
12	Yuxi, Yunnan province/Yan 2017 [47]	BA	2015–2016/County (secondary) hospital	Inpatient with new rural cooperative medical insurance/unreported	All patients (493groups)	DRGs payment in 9 hospitals from 2016	FFS payment in the same hospitals before the reform	Comparison of means before and after pilot, no formal test	1) expenditure per admission, 2) length of stay
13	Yuxi, Yunnan province/Zhou 2018 [48]	BA	Jan - Oct 2017/Tertiary hospital	Inpatient with urban employee or resident medical insurance/36,827 vs. unreported	All patients (531 groups)	DRGs payment in 1 hospital	FFS payment under global budget in the same hospital before the reform	Comparison of means, before (2016) and after pilot, no formal test	expenditure per admission

*CBA* controlled before after study, *BA* uncontrolled before-after study, *ITS* interrupted time series study, *DRGs* diagnosis related groups, *FFS* fee for service, *DID* difference-in-difference, *DDD* difference-in-difference-in-difference, *CV* coefficient of variation, ^a^The hospital grade system in mainland China has three major grade defined by the size, function, capability of clinical services of a hospital, from the highest to lowest are tertiary, secondary and primary care hospital, and within each grade there are three subgrade, from the highest to lowest are A, B and C. Tertiary A level hospitals usually have the highest capability of specialized care, the most advanced medical equipment and are mainly teaching hospitals with research responsibilities. Expenditure per admission = total expenditure of hospitalization/number of admitted patients

### Risk of bias assessment

Among the thirteen included studies, nine selected representative exposed cohort [11, 12, 14, 31, 43–47], eleven drawn control from the same community as the exposed group [11–14, 31, 41, 43, 45–48], only three considered comparability of groups on the basis of the design or analysis [11–13], five used appropriate statistical analysis [11–14, 31], and only two studies had low risk of bias in all items of the customized NOS (Table 2) [11, 12].

**Table 2:**
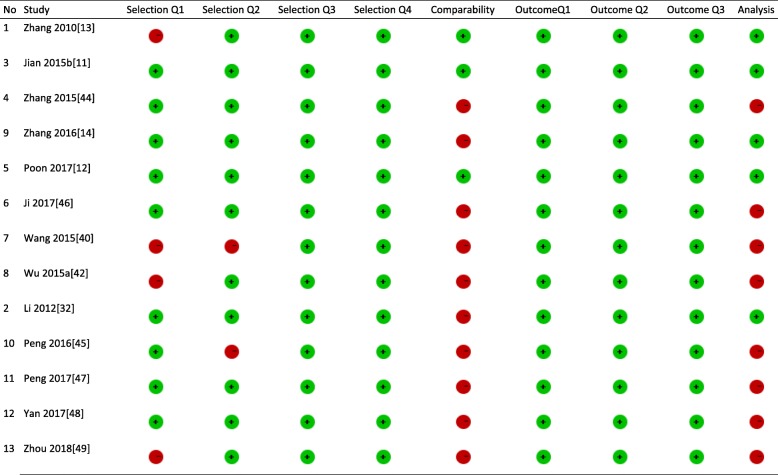
Quality assessment of included studies

unclear risk of bias,  high risk of bias,  low risk of bias, Selection Q1: Representativeness of the exposed cohort, Selection Q2: Selection of the non-exposed cohort, Selection Q3: Ascertainment of exposure, Selection Q4: Demonstration that outcome of interest was not present at start of study, Comparability: Comparability of cohorts on the basis of the design or analysis, Outcome Q1: Assessment of outcome, Outcome Q2: Was follow-up long enough for outcomes to occur, Outcome Q3: Adequacy of follow up of cohorts, Analysis: appropriate statistical analysis

### Expenditure of healthcare

The findings of the effect of DRGs payment on the EPA were mixed. Eight of the thirteen studies reported reduction of EPA after using DRGs payment, including four CBAs and four BAs [11, 12, 14, 31, 45–48]. However, one CBA reported that the EPA increased more in DRGs payment hospitals than controlled FFS payment hospitals [43], and two ITS and one BA found that the EPA increased after piloting DRGs payment [39, 41, 44], while one CBA reported that there was no significant difference of change between DRGs and FFS payment cases [13] (Table 3 and Additional file 1: Table S1).

**Table 3:** Summary of effects of diagnosis-related groups payment on healthcare in studies from mainland China

Location/Study	Study design	Expenditure per admission	Out of pocket payment	Length of stay	Quality of care	Equity of care	Up coding	Quality rating (NOS)*
Shanghai/Zhang 2010 [13]	CBA	↔		↔		↓		8
Beijing/Jian 2015b [11]	CBA	↓	↓	↔	↓	↓		9
Beijing/Zhang 2015 [43]	CBA	↑	↓	↓	↑	↓		7
Changsha, Hunan province/Zhang 2016 [14]	CBA	↓		↓	↑			8
Beijing /Poon 2017 [12]	CBA	↓			↓↑	↓		9
Beijing /Ji 2017 [45]	CBA	↓	↔	↓				7
Harbin, Heilongjiang province/Wang 2015 [39]	ITS	↑	↔	↔			↑	5
Guangxi province/Wu 2015a [41]	ITS	↑	↑	↔				6
Tianjin/Li 2012 [31]	BA	↓		↓	↑			8
Lufeng, Yunnan province/Peng 2016 [44]	BA	↑		↓			↑	6
Xiangyun, Yunnan province/Peng 2017 [46]	BA	↓		↓				7
Yuxi, Yunnan province/Yan 2017 [47]	BA	↓		↓				7
Yuxi, Yunnan province/Zhou 2018 [48]	BA	↓						6
Number of studies		13 (6 CBA, 2ITS, 5 BA)	5 (3 CBA, 2 ITS)	11 (5 CBA, 2 ITS, 4 BA)	5 (4 CBA, 1 BA)	4 CBA	1 ITS, 1 BA	
Summary of effect		Mixed	Mixed	Mild decrease	Mixed	Decrease	Increase	
Certainty of the evidence (GRADE)		Very low^1^	Very low^1^	Low^2^	Very low^1^	Moderate^3^	Low^2^	

High certainty: This research provides a very good indication of the likely effect. The likelihood that the effect will be substantially different† is low

Moderate certainty: This research provides a good indication of the likely effect. The likelihood that the effect will be substantially different† is moderate

Low certainty: This research provides some indication of the likely effect. However, the likelihood that it will be substantially different† is high

Very low certainty: This research does not provide a reliable indication of the likely effect. The likelihood that the effect will be substantially different† is very high

^1.^The evidence was by default graded as low as all studies were classified as non-randomised and observational studies, and further downgraded to very low due to the high risk of bias and inconsistency across findings

^2.^The evidence was by default graded as low as all studies were classified as non-randomised or observational studies

^3.^The evidence was by default graded as low as all studies were classified as non-randomised studies, but upgraded to moderate for consistency across findings

CBA: controlled before after study, ITS: interrupted time series study, BA: uncontrolled before-after study, *Number of items with low risk of bias in 9 total items of quality assessment using a modified Newcastle-Ottawa scale (NOS), Direction of change: ↑up, ↓down, ↔ even

The studies reported increase or no change of EPA were more likely to apply DRGs payment to a small groups of patients (13–36 groups of diseases), with only fixed rate for insurance payment but no clear policy incentives (such as fixed rate) to contain the overall EPA or OOP per admission [13, 39, 41], or allow hospitals to decide whether a patient is applicable to DRGs payment scheme [43].

The reported effects of DRGs payment on the OOP were also mixed (Table 3). Among the five studies, two CBA reported more reduction or smaller increase of OOP using DRGs payment than FFS [11, 43], one CBA and one ITS reported no difference between the two payment methods [39, 45], while one ITS reported an increase of OOP after using DRGs payment [41] (Table 3 and Additional file 1: Table S2).

### Efficiency of healthcare

Seven among the eleven studies reported that the LOS reduced after piloting DRGs payment, including three CBA and four BA [11–14, 31, 43–47]. However, two CBA and two ITS reported non-significant change comparing DRGs to FFS payment [13, 39, 41, 43], though the LOS actually slightly increased in the two ITS after piloting DRGs payment [39, 41] (Table 3 and Additional file 1: Table S3).

### Quality of healthcare

Five studies reported the impacts of DRGs payment on the quality of healthcare. On the positive side, one CBA in Beijing reported that the 2 weeks readmission rate reduced in DRGs payment piloting hospitals while increased in control hospitals (FFS) [43]. A CBA from Changsha found that the number of prescribed antibiotics and its expense was significantly lower in DRGs payment piloting hospitals than in FFS paid hospitals [14]. A BA in Tianjin found that the vaginal delivery rate of DRGs payment patients (55.9%) was significantly higher than that of FFS patients (22.7%) [31]. However, on the negative side, a CBA in Beijing found that the readmission rate increased more in DRGs payment hospitals (0.26%) than controlled FFS payment hospitals (0.13%) [11]. Another CBA in Beijing assessing the quality of care of acute myocardial infarction (AMI) found mixed results that the DRGs payment led to a 72.2% reduction of in-hospital mortality, but 7.1% reduction of the prescription of optimal AMI medications at arrival [12] (Table 3 and Additional file 1: Table S4).

### Equity of healthcare

Four CBA reported worrisome outcomes related to the equity of healthcare, including patient selection, cost shifting and worse outcomes in special patient groups [11–13, 43]. Two CBA in Beijing reported that more complicated or elderly DRGs eligible patients were reversed into FFS payment [11, 43]. Two CBA in Beijing also found that DRGs eligible but FFS payment cases had higher overall EPA [11, 12], higher OOP per admission [11], longer LOS [11, 12], higher readmission rate [11] and in-hospital mortality than DRGs payment inpatients [12]. What’s more, a CBA in Shanghai found cost shifting to uninsured patients [13] (Table 3 and Additional file 1: Table S5). These findings indicated that DRGs payment may impair the quality and equity of healthcare for certain groups of patients, especially those exempted from DRGs payment scheme, and highlighted the importance of measures to monitor and assure the quality and equity of healthcare.

### Provider behaviour change

One ITS and 1 BA found healthcare provider’s behaviour of up-coding of diagnoses or operations which tend to inflate the claims from the payers [39, 44] (Table 3).

### Sensitivity analysis

The results of the two studies with overall low risk of bias reported a reduction of EPA, OOP and LOS (though no statistical significance), but mixed results of quality of care and worsened equity of care, in which patient selection, cost-shifting, and longer LOS was found for patients who were exempted from DRGs payment scheme.

## Discussion

This review had five important findings. Firstly, there were large variations of DRGs payment policy design across studies in mainland China. When a study claimed experimented on DRGs payment, the patients involved may vary from several groups to all inpatients. There was also large variation in the method to determine the payment rate, whether a fixed rate of the overall EPA or OOP existed (compared to only a ceiling for the insurance reimbursement), whether hospitals can choose the payment method for an individual patient, which may create very different incentives to the behaviours of health providers.

Secondly, it seemed that DRGs payment was more likely to contain healthcare expenditure when it was consistently applied to all inpatients, with no differentiation by patient characteristics, disease status or insurance status, excepted for those proved to be more appropriate of other payment methods (such as per diem payment for long-term care or psychiatry diseases); when the base rate was calculated using historical data of the same level and type of hospitals, and involved negotiation between providers and insurance payers; with fixed rate of the overall expenditure (and OOP) per admission rather than for insurance payment only. Otherwise, DRGs payment may create unwanted incentives of providers to shift cost to OOP, uninsured, older or more complicated patients, which will harm the quality and equity of healthcare [11–13, 43].

Third, most studies indicated that DRGs payment could reduce the LOS, which is in accordance with findings from previous studies [6]. By reducing the LOS, the cost of per case will be decreased, and the efficiency will be increased, leading to higher productivity and profits for a hospital under per case payment system [17].

Fourth, we found that DRGs payment can improve the quality of healthcare by reducing unnecessary medications or procedures, such as the reduction of prescription of antimicrobials and caesarean rate [14, 31]. However, in some circumstances, optimal healthcare may be compromised, such as the reduction of prescription of optimal AMI medications at arrival of hospitals found in this review [12]. This indicated that quality assurance and monitoring mechanisms were vital as co-policies of DRGs payment system. Appropriate incentives to maintain or prompt high quality of healthcare may be considered, such as pay-for-performance or similar projects [49].

Fifth, as mentioned previously, the evidence of cost-shifting or patient selection was found, especially when the DRGs payment was only applied to a small number of groups (proportion) of patients,. There was cost-shifting to FFS paid, uninsured, older and more complicated cases [11–13, 43]. In mainland China, the hospital expenditure was consisted of insurance payment and OOP. And patients are usually requested to pay deposit when admission and can only get reimbursement when paying the whole hospitalization expenditure and discharged. There is also risk of cost-shifting to OOP when a ceiling was only applied to insurance payment but not to the total EPA or OOP, which may increase the financial burden of individual patients [9, 10].

Finally, we found up-coding behaviour of healthcare providers, a tempt and misconduct to locate the patient to a higher paid group than they should be, to obtain more profit [39, 44]. This indicated the necessary of regular checking and auditing of coding of medical records, in which rewards or penalties may be considered and applied to prompt upright conduct of healthcare providers.

There are few publications about the effects of DRGs payment on healthcare and patient outcomes in LMICs. A pervious SR included 12 studies from China, Thailand and Vietnam found that prospective payment had reduced health expenditure, LOS and readmission rates and improved service quality outcomes by reducing prescribing of unnecessary drugs and diagnostic procedures [23]. However, only 2 studies of DRGs payment (only one from China) was included in this review [11, 23]. Another SR examined the effects of “simplified DRGs payment” (so called ceiling price for a single disease) in China, concluded that the “simplified DRGs payment” could controlling hospitalization costs, but could not reduce LOS [24]. However, the so called “simplified DRGs payment” lacked of the grouping logics – a necessary component for DRGs payment, ignoring important patient characteristics such as age, gender, complications and surgical procedures, thus could hardly be recognized as real DRGs payment [24].

This review accumulated and critically evaluated current best available evidence of the effects of DRGs payment on healthcare in China, in which not only its desired effects but also unwanted effects were summarized. Important components of the DRGs payment policy design and their potential incentives were also discussed, which has consolidated and expanded the evidence base of the effects of DRGs payment on healthcare in LMICs [6]. Since China is a developing country, the evidence generated here may be helpful and adaptive to other developing countries with similar contexts and goals in health reform.

However, there were several limitations of this review. Firstly, though the largest number of eligible studies up-to-date were identified and included, some unpublished studies may be missing in this review. Secondly, the data extraction and quality assessment was conducted by one author, though double-checked, fell short of methodological rigours. Thirdly, no randomised trial has been found, and the quality of evidence for the majority of outcomes was generally low or very low. Fourthly, though important to understand the potential incentives and effects of DRGs payment, the details of components of DRGs payment policy and related context were under-reported in included studies, which may limit the interpretation and application of research findings. At last, only short time effects (1–2 years) of DRGs payment were identified in this review. Its long-term effects, especially on patient health outcomes, warrant further investigation.

## Conclusions

There is preliminary evidence that DRGs payment can mildly improve the efficiency of healthcare by reducing the LOS, but impair the equity and quality of healthcare, especially for those exempted from the DRGs payment scheme. However, DRGs payment may or may not containing the health expenditure, either total or OOP, depending on payment design. And its effects on quality of healthcare were mixed. What’s more, the strength of the evidence was limited by the low or very low overall quality for majority of the outcomes.

Health policy makers should be very careful in designing DRGs payment policy components, including using established sound DRGs grouping logics and appropriate payment rate calculation with reasonable adjustment, considering the settings (for example, secondary or tertiary hospitals) and range of implementation (for example, include all inpatients), and mechanisms to monitor and assure the quality and equity of healthcare.

Well-designed RCTs or other comparative studies measuring not only process outcomes but also patient health outcomes are warranted to consolidate the evidence base of DRGs payment for improving health system performance in China and other LMICs. To help interpreting findings and better inform health policy making, future studies explicitly describing the components of DRGs payment policy and contextual factors that may affect its effectiveness would be welcomed.

## Supplementary information

**Additional file 1: Table S1.** Summary of the effect of diagnosis-related groups payment on expenditure per admission (yuan)**. Table S2.** Summary of the effect of diagnosis-related groups payment on out-of-pocket payment (yuan). **Table S3.** Summary of the effect of diagnosis-related groups payment on length of stay (day). **Table S4.** Summary of the effect of diagnosis-related groups payment on quality of care. **Table S5.** Summary of the effect of diagnosis-related groups payment on equity of care.

## Data Availability

All data generated or analysed during this study are included in this manuscript.

## Abbreviations

AMI: Acute myocardial infarction
BA: Before after study
CBA: Controlled before after study
CNKI: Chinese National Knowledge Infrastructure
CV: Coefficient of variation
DRGs: Diagnosis related groups
EPA: Expenditure per admission
FFS: Fee-for-service
GRADE: Grading of Recommendations Assessment, Development and Evaluation
HICs: High income countries
ITS: Interrupted time series study
LMICs: Low- and middle- income countries
LOS: Length of stay
NRCMI: New rural cooperative medical insurance
NRCT: Non-randomised controlled trial
OOP: Out-of-pocket payment
PRISMA: Preferred Reporting Items for Systematic Reviews and Meta-Analyses
RCT: Randomised controlled trial
RRMI: Rural resident medical insurance
UEMI: Urban employee medical insurance
URMI: Urban resident medical insurance
