# Supplementary material for: The effects of diagnosis-related groups payment on hospital healthcare in China: a systematic review
Source: BMC Health Serv Res. 2020 Feb 12;20:112. doi: 10.1186/s12913-020-4957-5 (PMC7017558; doi:10.1186/s12913-020-4957-5)
Supplement: Supplementary file 1 — Additional file 1: Table S1. Summary of the effect of diagnosis-related groups payment on expenditure per admission (yuan). Table S2. Summary of the effect of diagnosis-related groups payment on out-of-pocket payment (yuan). Table S3. Summary of the effect of diagnosis-related groups payment on length of stay (day). Table S4. Summary of the effect of diagnosis-related groups payment on quality of care. Table S5. Summary of the effect of diagnosis-related groups payment on equity of care. [file 12913_2020_4957_MOESM1_ESM.docx]

**Supplementary**

**Search strategy in PubMed**

#1 "Diagnosis-Related Groups"[Mesh]

#2 "diagnosis related groups"

#3 "diagnosis related group"

#4 "China"[Mesh] or China

#5 #1 or #2 or #3

#6 #4 and #5

Table S1 Summary of the effect of diagnosis-related groups payment on expenditure per admission (yuan)

| Study | Location | Study design | Comparison | DRGs | FFS | Coefficients/ I-C | test | p | Effect |
| --- | --- | --- | --- | --- | --- | --- | --- | --- | --- |
| Zhang 2010 | Shanghai | CBA | Change |  |  | -0.278 | DDD | >0.05 | ⭤ |
| Jian 2015 | Beijing | CBA | Change | -62 | 109 |  | DID | <0.01 | ⭣ |
| Zhang 2015 | Beijing | CBA | Change | 457 | 180 |  |  |  | ⭡ |
| Zhang 2016 | Changsha, Hunan province | CBA | Endpoint | 2518 | 4484 |  | Student’s t-test | 0.000 | ⭣ |
| Ji 2017 | Beijing | CBA | Change | -0.07 | 0.05 |  |  |  | ⭣ |
| Poon 2017 | Beijing | CBA | Change |  |  | -0.241 | DID |  | ⭣ |
| Wang 2015 | Harbin, Heilongjiang province | ITS | ITS | 168.82* | -8.13* | 176.95* | ITS | 0.000 | ⭡ |
| Wu 2015a | Guangxi province | ITS | ITS | 14.48* | -0.31* | 380 | ARIMA |  | ⭡ |
| Li 2012 | Tianjin | BA | Endpoint | 6049 | 6891 |  | Mann- Whitney U | <0.01 | ⭣ |
| Peng 2016 | Lufeng, Yunnan province | BA | Endpoint | 2764 | 2467 |  |  |  | ⭡ |
| Peng 2017 | Xiangyun county, Yunnan province | BA | Endpoint | 3614 |  | -21 |  |  | ⭣ |
| Yan 2017 | Yuxi, Yunnan province | BA | Endpoint | 4115 | 4243 |  |  |  | ⭣ |
| Zhou 2018 | Yuxi, Yunnan province | BA | Endpoint | 10573 ^a^ |  | -1999 ^a^ |  |  | ⭣ |
|  |  |  | Endpoint | 9370 ^b^ |  | -260 ^b^ |  |  |  |

DRGs: diagnosis-related groups payment, FFS: fee for service payment, CBA: controlled before after study, CBA: controlled before after study, ITS: interrupted time series study, BA: uncontrolled before-after study, I-C: the difference between intervention and control group, DDD: difference-in-difference-in-difference, DID: difference-in-difference,  ^a^ urban employee medical insurance, ^b^ urban resident medical insurance, ITSA: Interrupted time series analysis, ARIMA: Autoregressive Integrated Moving Average model, ⭡ up, ⭣down, ⭤ even, * per month

Table S2 Summary of the effect of diagnosis-related groups payment on out-of-pocket payment (yuan)

| Study | Location | Study design | Comparison | DRGs | FFS | Coefficients/I-C | test | p | Effect |
| --- | --- | --- | --- | --- | --- | --- | --- | --- | --- |
| Jian 2015b | Beijing | CBA | Change | -387 | -237 |  | DID | <0.01 | ⭣ |
| Zhang 2015 | Beijing | CBA | Change | 111 | 220 |  |  |  | ⭣ |
| Ji 2017 | Beijing | CBA | Endpoint | 47.3% | 45.2% |  |  |  | ⭤ |
| Wang 2015 | Harbin, Heilongjiang province | ITS | ITS | 58.22* | -40.3* | 98.09* | ITS | 0.000 | ⭡ |
| Wu 2015a | Guangxi province | ITS | ITS | 3.28 * | -53.56* | 311 | ARIMA |  | ⭡ |

DRGs: diagnosis-related groups payment, FFS: fee for service payment, CBA: controlled before after study, , ITS: interrupted time series study, I-C: the difference between intervention and control group, DID: difference-in-difference, ARIMA: Autoregressive Integrated Moving Average model, ⭡ up, ⭣down, ⭤ even, * per month

Table S3 Summary of the effect of diagnosis-related groups payment on length of stay (day)

| Study | Location | Study design | Comparison | DRGs | FFS | Coefficients/I-C | test | p | Effect |
| --- | --- | --- | --- | --- | --- | --- | --- | --- | --- |
| Zhang 2010 | Shanghai | CBA | Change |  |  | -0.1481 | DDD | >0.05 | ⭤ |
| Jian 2015b | Beijing | CBA | Change | -1.5 | -1.5 |  | DID | >0.05 | ⭤ |
| Zhang 2015 | Beijing | CBA | Change | -0.67 | -0.78 |  |  |  | ⭣ |
| Ji 2017 | Beijing | CBA | Endpoint | 6.2 | 7.3 |  | Student’s t-test | <0.05 | ⭣ |
| Poon 2017 | Beijing | CBA | Change |  |  | -15.40% | DID |  | ⭣ |
| Wang 2015 | Harbin, Heilongjiang | ITS | ITS | 0.07* | 0.04* | 0.03* | ITS | 0.737 | ⭤ |
| Wu 2015a | Guangxi | ITS | ITS | 0.053* | -0.078* | -0.538 | ARIMA |  | ⭤ |
| Li 2012 | Tianjin | BA | Endpoint | 5.6 | 8.2 |  | t test | <0.01 | ⭣ |
| Peng 2016 | Lufeng, Yunnan province | BA | Endpoint | 6.7 | 7.6 |  |  |  | ⭣ |
| Peng 2017 | Xiangyun county, Yunnan province | BA | Endpoint | 6.41 |  | -0.22 |  |  | ⭣ |
| Yan 2017 | Yuxi, Yunnan province | BA | Endpoint (range) | 6.19-9.15 | 6.22-9.53 |  |  |  | ⭣ |

DRGs: diagnosis-related groups payment, FFS: fee for service payment, CBA: controlled before after study, ITS: interrupted time series study, BA: uncontrolled before-after study, I-C: the difference between intervention and control group, ITSA: Interrupted time series analysis, ARIMA: Autoregressive Integrated Moving Average model, ⭡ up, ⭣down, ⭤ even, * per month

Table S4 Summary of the effect of diagnosis-related groups payment on quality of care

| Study | Location | Study design | Outcome | Comparison | DRGs | FFS | test | p | Effect |
| --- | --- | --- | --- | --- | --- | --- | --- | --- | --- |
| Jian 2015b | Beijing | CBA | Readmission rate | Change | 0.26% | 0.13% |  |  | ⭣ |
| Zhang 2015 | Beijing | CBA | 2 weeks readmission rate | Change | -0.14% | 1.48% |  |  | ⭡ |
| Poon 2017 | Beijing | CBA | In-hospital mortality | Change | -72.2% |  | DID |  | ⭡ |
|  |  |  | Prescription of optimal AMI medications at arrival | Change | -7.1% |  | DID |  | ⭣ |
| Zhang 2016 | Changsha, Hunan province | CBA | Number of prescribed antibiotics | Endpoint | 2.41 | 3.04 | t test | 0.001 | ⭡ |
|  |  |  | Expenditure on antibiotics, yuan | Endpoint | 476 | 1108 |  |  | ⭡ |
| Li 2012 | Tianjin | BA | Vaginal delivery rate | Endpoint | 55.9% | 22.7% | χ2 | <0.01 | ⭡ |

DRGs: diagnosis-related groups payment, FFS: fee for service payment, CBA: controlled before after study, BA: uncontrolled before-after study, I-C: the difference between intervention and control group, ⭡ up, ⭣down, ⭤ even

Table S5 Summary of the effect of diagnosis-related groups payment on equity of care

| Study | Location | Study design | Outcome | DRGs payment | DRG eligible but FFS payment | Coefficients | test | p | Effect |
| --- | --- | --- | --- | --- | --- | --- | --- | --- | --- |
| Zhang 2010 | Shanghai | CBA | Expenditure per admission |  |  | -0.3357* | DDD | <0.05 | ⭣ |
| Jian 2015b | Beijing | CBA | Expenditure per admission, yuan | 19671 | 20946 |  |  |  | ⭣ |
|  |  |  | Out of pocket expenditure, yuan | 5418 | 5830 |  |  |  | ⭣ |
|  |  |  | Length of stay, day | 8.9 | 10.0 |  |  |  | ⭣ |
|  |  |  | Readmission rate | 2.8% | 6.8% |  |  |  | ⭣ |
| Zhang 2015 | Beijing | CBA | Patient selection |  | more complicated cases reversed to FFS payment, texts description only |  |  |  | ⭣ |
| Poon 2017 | Beijing | CBA | Expenditure per admission increase, % |  |  | 24.1% |  |  | ⭣ |
|  |  |  | Length of stay increase, % |  |  | 15% |  |  | ⭣ |
|  |  |  | In-hospital mortality increase, % |  |  | 5.4% |  |  | ⭣ |

DRGs: diagnosis-related groups payment, FFS: fee for service, CBA: controlled before after study, * the difference between DRG eligible but FFS payment cases and DRGs payment cases, ⭡ up, ⭣down, ⭤ even.
